# Outcomes after Surgical Treatment for Rectal Atresia in Children: Is There a Preferred Approach? A Systematic Review

**DOI:** 10.1055/s-0042-1758152

**Published:** 2022-12-14

**Authors:** Cunera M. C. de Beaufort, Joep P. M. Derikx, Justin R. de Jong, George L. Burchell, Sterre R. J. Bosscha, Sjoerd A. de Beer, Lodewijk W. Ernest van Heurn, Ramon R. Gorter

**Affiliations:** 1 Department of Pediatric Surgery, Emma Children's Hospital Amsterdam University Medical Center, University of Amsterdam, Amsterdam, the Netherlands; 2 Amsterdam Gastroenterology and Metabolism Research Institute, Amsterdam, the Netherlands; 3 Amsterdam Reproduction and Development Research Institute, Amsterdam, the Netherlands; 4 Department of Medical Library, Amsterdam University Medical Center, Vrije Universiteit of Amsterdam, Medical Library, Amsterdam, the Netherlands

**Keywords:** rectal atresia, children, surgical intervention, pull-through procedure, posterior sagittal anorectoplasty

## Abstract

Rectal atresia (RA) affects only 1 to 2% of all children with anorectal malformations. No consensus on optimal treatment strategy is yet achieved. Therefore, the aim of this systematic review is to summarize all surgical interventions for RA and outcomes described in the current literature. A literature search was conducted in PubMed, Embase, Web of Science, and Cochrane Library on January 24, 2022. All studies describing treatment for RA in children (< 18 years) were included. Operation technique and postoperative complications were listed. Only descriptive analysis was anticipated. Quality of the studies was assessed using Johanna Briggs Institute critical appraisal checklist for case reports and series. The search yielded 6,716 studies of which, after duplicate removal, 4,028 were excluded based on title and abstract screening. After full-text assessment, 22 of 90 studies were included, yielding 70 patients. Posterior sagittal anorectoplasty (PSARP) and pull-through were most performed (43/70 and 18/70 patients, respectively). Four patients experienced postoperative complications: anal stenosis ( *n* = 1), anastomotic stenosis ( *n* = 2), and death due to a pulmonary complication ( *n* = 1). In the low-quality literature available, most patients with RA are treated with PSARP or pull-through technique. A low complication rate of both has been described but follow-up was often not mentioned. Larger well-designed studies should be performed to determine optimal treatment strategy for children with RA. This study reflects level of evidence V.

## Introduction

Anorectal malformations (ARM) occur in approximately 1 per 5,000 live births each year. 1 An extremely rare entity amongst ARM is rectal atresia (RA), representing approximately 1to 2% of all ARM 1 and is, according to the Krickenbeck classification for ARM, therefore categorized under the “rare” variants. 2 3 RA can be subdivided into five types with potentially heterogeneous disease morphology and clinical presentation. 4 These five types of RA are as follows: type I: rectal stenosis, (A) intramural, (B) web with a hole; type II: RA with a septal defect; type III: RA with a fibrous cord between two atretic ends; type IV: RA with a gap; type V: multiple: (A) RA with stenosis, (B) multiple RA, and (C) thickened Houston's valves/multiple rectal stenosis; as illustrated by Sharma and Gupta 4 ).

Children with RA require surgical treatment due to the clinical consequences of this disease (e.g., inability to pass meconium, bowel distension, and potentially sepsis). In most children, initially a diverting (sigmoid) colostomy is created as prompt surgical intervention, after which at later stage, a definitive reconstructive intervention is performed. 5 Definitive reconstruction in children with RA can be done using several different surgical techniques. For example, local excision (e.g., opening the rectal web, stricturoplasty, dilation, and perforation), anorectoplasty (dissecting posterior sphincter complex or both posterior and anterior), pull-through surgery (e.g., transanal endorectal pull-through [similar to surgical treatment for Hirschsprung's disease (HD)] 6 ), abdominoperineal pull-through, sphincter saving pull-through), and other innovative treatment options (e.g., magnamosis). 7 Some techniques are rarely used and relatively new, such as magnamosis, a treatment strategy that involves the use of two magnets to create an anorectal anastomosis (previously described as treatment for esophageal atresia 8 ). However, no consensus has yet been reached on the optimal treatment strategy because different RA types may necessitate different treatment strategies or surgical approaches.

Due to the rarity of RA and its heterogeneous presentation, lack of clarity exists on what the optimal treatment strategy and their outcomes is. Case reports and series describing techniques for treatment of RA have been published. Yet, the current literature lacks a systematic review. Therefore, the aim of this systematic review is to provide an overview of the surgical interventions used for RA and their outcomes in the current available literature.

## Materials and Methods

### Study Design

A systematic review was performed according to the Preferred Reporting Items for Systematic Reviews and Meta-analyses (PRISMA) statement guidelines ( Supplementary Table A1 , available in the online version only). 9 This review has been registered at the International Prospective Register of Systematic Reviews (PROSPERO, registration number: 309464).

### Literature Search

A systematic literature search was performed in PubMed (Medline), Embase.com (Ovid), Clarivate Analytics/Web of Science Core Collection, and the Wiley/Cochrane Library. The timeframe within the databases was from inception to January 24, 2022. The search strategy was developed in collaboration with a clinical librarian (G.L.B.). The search included keywords and free text terms for (synonyms of) “Rectal Atresia” OR “Anorectal malformation,” combined with (synonyms of) “Pull-through” OR “Surgery.” Studies were also cross-referenced for additional articles. A full overview of the search terms per database can be found in the Supplementary Table A2 (available in the online version only).

### Eligibility Criteria

Studies were included if they described the surgical treatment for RA in the pediatric population (< 18 years of age) or if the data on RA could be separately extracted from any other population described in the article. Randomized controlled trials (RCT's), retrospective or prospective cohort studies, case series, and case reports were eligible for inclusion. Studies were excluded if they described other types of rare ARM or ARM in general, prenatal/fetal surgical treatment of RA, or adults who had surgical correction of RA in childhood, if they were published before January 1, 2000, or in languages other than English or Dutch, if studies were unpublished or only abstract were available.

### Study Selection and Methodological Quality Assessment

After duplicate removal, two reviewers (S.R.J.B. and C.M.C.B.) independently screened all titles and abstracts using Rayyan. 10 Studies were obtained in full-text and further selected based on full text. In case, articles were not available in full text, authors were contacted. In case of disagreement between reviewers, a third reviewer (R.R.G.) was consulted for final decision.

Two independent reviewers (S.R.J.B. and C.M.C.B.) assessed methodological quality, using the Joanna Briggs Institute (JBI) Critical Appraisal Checklist for Case reports and Case series. 11 12 For case reports, this tool comprises eight questions regarding methodological quality with four answer options (“Yes,” “Unclear,” “Not applicable,” or “No”; Supplementary Document 1 [available in the online version only]). Each answer option was quantified by assigning a score of 3, 2, 1, or 0, respectively. Scores were summed, with a maximum of 24 points for each case report. “High” quality was defined as 19 points or higher, “Moderate” as 14 to 18 points, and “Low” defined as 13 points or fewer. For case series, this tool comprises 10 questions regarding methodological quality with the four answer options ( Supplementary Document 2 , available in the online version only). A maximum score of 30 could be assigned. “High” quality was defined as 24 or higher, “Moderate” as 17 to 23, and “Low” as 16 or fewer. In case of cohort studies, methodological quality was assessed by using the Newcastle–Ottawa Scale (NOS). 13 In case of disagreement, a third reviewer (R.R.G.) was consulted for final decision.

### Data Extraction

Data on identification features of the study, study characteristics, participant characteristics, associated anomalies (i.e., cardial, tracheoesophageal, and urological anomalies; presacral mass; spinal anomalies; and tethered cord), type of RA (defined as reported by the original study), type of surgical intervention, and results were extracted by two independent reviewers (S.R.J.B. and C.M.C.B.) according to predefined data collection forms from all included studies. Definitive surgical intervention was categorized into four main treatment groups (i.e., PSARP (posterior sphincter complex dissected or both posterior and anterior), pull-though (i.e., transanal, abdominoperineal, laparoscopic assisted, and sphincter saving), local excision (i.e., transanal, stricturoplasty, opening rectal web, and endoscopic) and other (i.e., magnamosis). In case of uncertainties, a third reviewer (R.R.G.) was consulted for final decision.

### Outcomes

Primary outcomes were the number of surgical interventions and the number of postoperative complications after definitive operation technique (i.e., postoperative complications within 30 days [defined as reported by the original study (i.e., anal or rectal stricture, wound infection, wound dehiscence, and rectal prolapse)]). Secondary outcomes were severity of complications according to Clavien–Dindo grading 14 and functional outcomes at follow-up with time points as reported by the original study (i.e., constipation, soiling, urinary continence, voiding difficulties, and sexual development) and quality of life. Functional bowel outcomes (e.g., voluntary bowel movements, soiling, and constipation) were assessed according to the Holschneider continence score. 15 If postoperative complications or functional outcomes were not explicitly described in the original study, it was categorized as not present, and authors were contacted for missing data. If additional information was provided, it was incorporated in the results section. If additional data were not provided, it was categorized as missing data.

### Statistical Analysis

Descriptive statistics were used for analysis of baseline, disease, and treatment characteristics. These were reported as proportions for binary or categorical variables. Missing or unknown data were described. No comparisons, quantitative syntheses, or meta-analyses were performed due to the expected small numbers and large heterogeneity.

## Results

### Study Selection

**Fig. 1:**
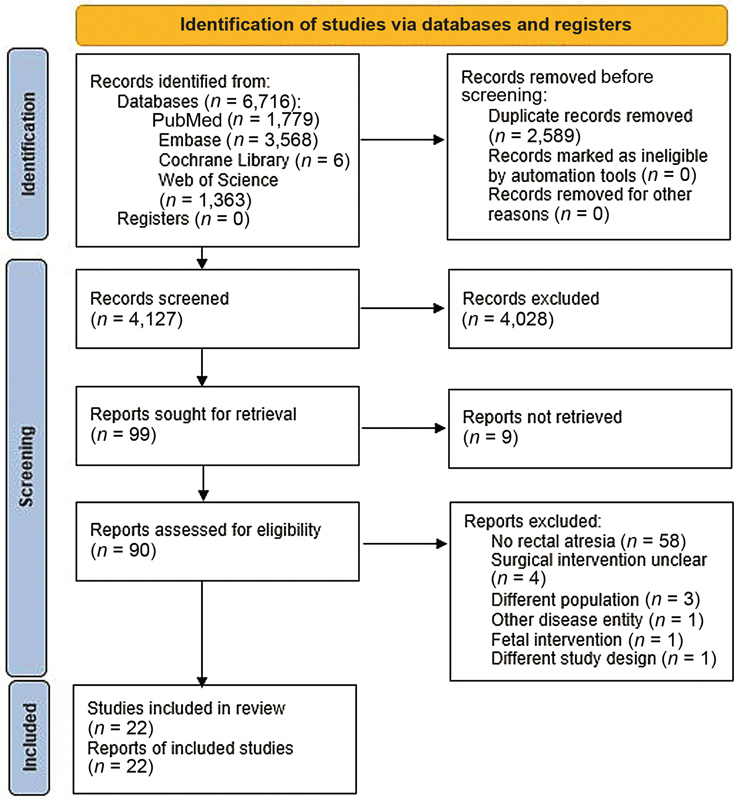
PRISMA 2020 flow diagram. PRISMA, Preferred Reporting Items for Systematic Reviews and Meta-analyses.

In total, 6,716 studies were identified through searching PubMed ( *n* = 1,779), Embase ( *n* = 3,568), Web of Science ( *n* = 1,363), and Cochrane Library ( *n* = 6). After duplicate removal, the remaining 4,127 studies were screened on title and abstract. Ninety-nine studies were eligible for full-text assessment of which 22 were included in this systematic review. In nine studies, full text was not available and authors were contacted of whom none responded. The PRISMA flowchart of study selection with reasons for exclusion is shown in Fig. 1 .

In total, 77 studies were excluded of which the following of interest: four studies with reason “surgical intervention unclear” as RA was solely mentioned in the study without describing surgical treatment. 2 16 17 18 Furthermore, three studies were excluded based on “different population” due to adult patient in one study 19 and data on RA not separately extractable from the ARM population in two studies. 20 21 Finally, one study was excluded with reason “other disease entity,” due to RA occurring after necrotizing enterocolitis (NEC), 22 and one study with reason “fetal intervention” due to RA correction in utero. 23

### Study Characteristics

In total, four case series and 18 case reports were included, comprising a total of 70 patients with RA (37 males, 21 females, and 12 unknown 4 5 7 24 25 26 27 28 29 30 31 32 33 34 35 36 37 38 39 40 41 42 ). Age at definitive surgical correction ranged from 1 day (after full-term gestational age) to 6 years of age. Table 1 shows characteristics of the 22 included studies and their included patients. None of the included studies have used the classification of RA by Sharma and Gupta, 4 since the introduction in 2017. Unfortunately, due to the unclear reporting of RA definition, classification accordingly was not possible. Various definitions of RA have been used by the included studies as depicted in Supplementary Table A3 (available in the online version only).

**Table 1:** General characteristics of included studies and patients

Study	Year	Country	Study design	Number of patients	Sex	Age at definitive surgery	Surgical technique	Follow-up
Ahn et al 24	2019	The United States	Case report	1	M	11 months	Pull-through	2 months
Gieballa et al 5	2018	Saudi Arabia	Case report	3	M	6–10 months	Pull-through ( *n* = 3)	4 years
								4 years
								4 years
Mehmetoğlu 25	2018	Turkey	Case report	1	M	9 months	PSARP a	4,5 years
Sharma and Gupta 4	2017	India	Case series	10	7 M, 3 F	Unknown	PSARP d ( *n* = 4), Pull-through ( *n* = 2), Local excision ( *n* = 4)	3 years
Braiek et al 26	2016	Tunisia	Case report	1	M	2 days	Local excision	6 months
Lane et al 27	2016	The United States	Case report	1	M	10 months	PSARP a	Unknown
Eltayeb and Shehata 28	2015	Egypt	Case series	2	1 M, 1 F	8–9 months	Pull-through ( *n* = 2)	18 months
								18 months
Laamrani and Dafiri 29	2014	Morocco	Case report	1	F	20 days	PSARP b	Unknown
Russell et al 7	2014	The United States	Case report	1	M	4 months	Magnamosis	4 years
Hamrick et al 30	2012	The United States	Case series	17	10 M, 7 F	2 days–6 years	PSARP a ( *n* = 17)	4 years
Hamzaoui et al 31	2012	Tunisia	Case report	1	F	6 months	Pull-through	2 years
Stenström et al 32	2011	Sweden	Case report	1	F	4 months	Local excision	3 months
Hosseini et al 33	2009	Iran	Case report	1	F	2 years	Pull-through	Unknown
Luo et al 34	2009	Taiwan	Case report	3	F	3–6 months	PSARP b ( *n* = 1), Pull-through ( *n* = 2)	3 years
								1 year
								3 months
Lee et al 35	2007	China	Case report	1	M	5 months	PSARP b	2 years
Nguyen and Pham 36	2007	Vietnam	Case report	2	F	3 months	Pull-through ( *n* = 2)	8 months
								5 months
Ibrahim 37	2006	Egypt	Case report	4	3 M, 1 F	1–5 days	Pull-through ( *n* = 4)	Unknown
								Unknown
								Unknown
								Not applicable e
Kisra et al 38	2005	Morocco	Case report	4	M	3 months	PSARP b ( *n* = 4)	Unknown
								Unknown
								11 years
								15 years
Kobayashi et al 39	2005	Japan	Case report	1	M	4 months	PSARP b	5 years
Belizon et al 40	2005	The United States	Case series	12	Unknown	Unknown	PSARP d	Unknown
Saxena et al 41	2004	Germany	Case report	1	M	1 day	Local excision	Unknown
Ramesh et al 42	2002	Malaysia	Case report	1	M	4 weeks	PSARP d	9 months

Abbreviations: F, female; M, male; PSARP, posterior sagittal anorectoplasty.

a Posterior sphincter opened

b Posterior and anterior sphincter opened.

c Anterior sphincter opened.

d Sphincter opening not mentioned.

e Not applicable because of death due to pulmonary complication.

### Methodological Quality

According to the JBI Critical Appraisal Checklist, 10 of 18 case reports were of high quality with a score of 19 or greater. 5 7 32 33 34 36 37 38 41 42 The remaining 8 of 18 were of moderate quality with scores ranging from 16 to 18. 24 25 26 27 29 31 35 39 Moderate scores were mostly due to lack of description of adverse or unanticipated events and no provision of takeaway lessons. Of the case series, two of four studies were of high quality with scores of 25 and 28, respectively. 4 30 The other two of four studies were of moderate quality with scores of 20 and 22, respectively. 28 40 No studies were of low quality. An overview of individual methodological quality assessment of included studies is provided as Supplementary Table A4 (available in the online version only).

### Definitive Operation Technique and Number of Postoperative Complications

In general, 16 of 22 studies reported postoperative follow-up less than 30 days for (some) included patients, comprising 56 of 70 patients. In 4 of 56 patients, a postoperative complication was noted (i.e., anal stenosis [ *n* = 1], anastomotic stenosis [ *n* = 2], and death [ *n* = 1]). 4 5 7 24 25 26 28 30 32 34 36 37 38 39 40 41 Clavien–Dindo scores per postoperative complication are shown in Table 2 .

**Table 2:** Treatment options and postoperative complications

Surgical intervention	Number of patients	Reporting follow-up < 30 days a *n* (%)	Postoperative complications b	Classification c	Number of studies
Posterior sagittal anorectoplasty	43	37 (86.0)	Anal stenosis ( *n* = 1) 30 Anastomotic stenosis ( *n* = 2) 38	IIIa IIIa	10 4 25 27 29 30 34 35 38 39 40
Pull-through					
Transanal	10	5 (50.0)	Death ( *n* = 1) 37	V	4 5 31 34 37
Laparoscopic assisted	3	2 (66.7)	–	–	2 24 36
Abdominoperineal	3	2 (66.7)	–	–	2 4 33
Sphincter saving	2	2 (100.0)	–	–	1 28
Local excision					
Transanal	4	4 (100.0)	–	–	2 4 41
Opening rectal web	2	1 (50.0)	–	–	2 26 42
Stricturoplasty	1	1 (100.0)	–	–	1 4
Endoscopic	1	1 (100.0)	–	–	1 32
Other					
Magnamosis	1	1 (100.0)	–	–	1 7

a Number of patients with adequate reporting of follow-up < 30 days.

b Postoperative complication < 30 days.

c Classification according to Clavien–Dindo guidelines for postoperative surgical complications. 14

In total, four main groups of treatment strategy were reported of which PSARP and pull-through approach were performed most often in 43 of 70 patients and 18 of 70 patients, respectively ( Table 2 ).

Regarding PSARP ( *n* = 43), data on postoperative complications were present in 38 patients of whom 3 experienced a postoperative complications (i.e., two anastomotic stenosis which required dilatation for 3 months and one anal stenosis requiring dilatations; Table 2 ). 30 38

Regarding pull-through surgery (transanal, laparoscopic assisted pull-through, abdominoperineal pull-through, and sphincter saving pull-through surgery, *n* = 18), data on postoperative complications were present in 11 patients of whom 1 experienced a postoperative complication after transanal pull-through surgery (death due to clinical deterioration and pulmonary complications; Table 2 ). 37 No other postoperative complications occurred.

Regarding local excision (i.e., transanal, opening rectal web, stricturoplasty, or endoscopic, *n* = 8), data on postoperative complications were present in seven patients of whom none experienced postoperative complications. Regarding magnamosis ( *n* = 1), no postoperative complications occurred at follow-up of less than 30 days.

Details of the used operative techniques and their postoperative complications (according to Clavien-Dindo) are demonstrated in Table 2 . Further specification of used operative technique (e.g., dissection of sphincter complex and preservation of anal canal) is shown in Supplementary Table A3 (available in the online version only).

### Definitive Operation Technique and Functional Outcomes

In general, 18 of 22 studies reported functional outcomes for (some) included patients, comprising 52 of 70 patients. 4 5 7 24 25 26 28 30 31 32 34 35 36 37 38 39 41 42 For bowel and urinary function, follow-up ranged from 2 months to 15 years after definitive correction of RA ( Table 1 ). However, timing of follow-up was not mentioned in three studies, comprising four patients. 37 38 41

Regarding PSARP ( *n* = 43), data on functional outcomes were available for 28 of 43 patients, regarding constipation/soiling. Constipation (grade IIA, requiring laxative treatment) occurred in six of them (at follow up 48–54 months). 25 30 The remaining follow-up in other studies ranged from 9 months to 5 years ( Table 1 ). Occasional soiling occurred in one patient (at 3-year follow-up). 34 The remaining 21 patients had daily stool and voluntary bowel movements (grade I). No studies describing PSARP reported data on functional urinary outcomes.

Regarding pull-through surgery ( *n* = 18), data on functional outcomes were available for 15 of 18 patients regarding constipation/soiling, and for 1 of 18 patients regarding urinary outcomes. In total, 5 of 15 patients developed constipation (grade IIA) (three after transanal pull-through, one after abdominoperineal pull-through, and one after sphincter saving pull-through) at, respectively, 18 months, 3, and 4 years of follow-up. 4 5 28 No soiling was reported. One case was found to have neurogenic bladder disorder after presenting at 1 year follow-up with a febrile urinary tract infection and grade II vesicoureteral reflux bilaterally, requiring clean intermittent catheterization (CIC) and prophylactic antibiotics up until he reached the age of 12 years. 5

Regarding local excision ( *n* = 8), data on functional outcomes were available for 8 of 8 patients of whom all had normal bowel function at follow-up ranging from 3 months to 3 years. No data on urinary outcomes were reported.

The one patient that underwent magnamosis had normal bowel function at 4 years of follow-up. 7 No data on urinary outcomes were reported.

### Sexual Development and Quality of Life

No studies reported data on sexual development or quality of life at follow-up.

### Diversion Colostomy

The use of a diverting (sigmoid) colostomy was reported for 56 of 70 patients of whom 45 of 56 had a (sigmoid) colostomy. Timing on colostomy placement differed between directly at presentation (after birth; 17/45) or during definitive RA correction (28/45). Different types of colostomy were performed (e.g., left transverse loop colostomy, right transverse loop colostomy, double barrel sigmoid colostomy, and sigmoid colostomy). Colostomy was temporary in all patients as all patients underwent definitive surgical correction of RA.

### Associated Anomalies

In 45 of 70 patients, it was reported whether associated anomalies were present and in 6 of 45 anomalies were identified. Regarding patients who underwent PSARP, 4 of 43 had associated anomalies (i.e., balanic hypospadias [ *n* = 1], ectopic kidney [ *n* = 1], tracheoesophageal fistula [ *n* = 1], and bilateral nonpalpable cryptorchidism [ *n* = 1]). 4 30 38 Regarding patients who underwent (transanal) pull-through, 1 of 18 had congenital heart disease, minor omphalocele, hypospadias, and undescended testes. 5 Regarding patients who underwent (transanal) local excision, one of eight had a congenital heart disease. 41 In 30 of 70 patients, it was reported whether presacral mass was present and in 8 of 30 presacral mass was identified of whom all underwent resection during their definitive operation (PSARP). 30

## Discussion

RA is extremely rare and little is known about the different treatment options. This systematic review of the available literature showed that only low-quality data (case series and case reports) are available, with the majority of patients (61/70) undergoing either posterior sagittal or pull-through approach. Postoperative follow-up of less than 30 days was reported in 38 of 43 patients for PSARP and in 11 of 18 patients for pull-through, and postoperative complications occurred in 3 of 38 patients and 1of 11 patients, respectively. Functional outcomes were reported in 28 of 43 patients for PSARP and 15 of 18 patients for pull-through, with constipation in 6 of 28 patients and 5 of 15 patients, respectively.

This study found data reported on postoperative complications in 57 of 70 patients, with postoperative complications occurring in 4 of 57 patients. A previous systematic review, comparing laparoscopic assisted anorectal pull-through (LAARP) and PSARP in children with high and intermediate ARM, found a higher overall complication rate of 28% (23% for LAARP and 33% for PSARP) with a slight lower reporting rate of 60%. 43 Unfortunately, in contrast to our study, postoperative complications were not further specified. In our study, postoperative complications can be underreported in the included studies as follow-up of less than 30 days was not reported in 13 of 70 patients. In addition, one study 40 only reported on rectal prolapse and did not mention if other possible postoperative complications were investigated. This registration and reporting bias likely leads to an underestimation of postoperative complications and therefore a too positive view of the outcomes in this cohort. The same goes for functional outcomes, as one-fifth of the studies did not report this. In addition, data on bowel continence were missing in a quarter of the patients, data on urinary outcomes were only reported in 1 of 70 patients, and no data on sexual development and quality of life were reported. This could be partially explained by the fact that most included studies had a follow-up of 4 years or lesser and included children (≤ 5 years of age) who might not yet be potty-trained or are sexually active. Urinary and quality of life outcomes might be difficult to assess in the early follow-up. These data demonstrate the importance, especially in research for rare diseases, of (future studies with) uniformity and adequate definitions and reporting of postoperative complications and functional outcomes. Clear core outcome set developed in conjunction with the entire multidisciplinary team and patient representatives might be the solution.

Children with RA most often have a normal positioned anus, with an atretic segment approximately 2 cm above the dentate line. On one hand, by using pull-through technique, the sphincter complex on the posterior side remains unaffected and intact with minimal scarring of healthy tissue as a result. However, in the presence of a presacral mass, pull-through surgery cannot be performed because of this anatomical variation. On the other hand, PSARP (dissecting the posterior side or the complete sphincter [posterior as well as anterior side]) may be preferred, as pull-through surgery (e.g., such as in HD) can lead to soiling or fecal incontinence due to overstretching the sphincter complex. In addition, in case of a long atretic segment, the urethra or vagina could be at risk for damage during surgery. A meta-analysis on postoperative outcomes in HD after transanal endorectal pull-through showed that the mean incidence of constipation was 9.0% of incontinence or soiling was 6.3%, and of anastomotic stricture was 11%, with duration of follow-up ranging from 12 to 60.5 months. 44 In our study, this was 33.3, 0.0, and 0.0%, respectively, with duration of follow-up ranging from two to 180 months. These differences may be caused by different disease etiology between HD and RA, heterogeneous patient groups, various procedures in our population (i.e., not only pull-through techniques), and registration and publication bias. Four different treatment strategies (PSARP, pull-through, local excision, and other) were described in studies published from 2000 onwards of which PSARP and pull-through techniques were the majority. Within the four main treatment strategy groups, large heterogeneity in surgical technique was found. For example, in the PSARP group, in some patients, only the posterior side of the sphincter complex was dissected, as in others the complete sphincter complex was dissected (posterior and anterior; as depicted in Supplementary Table A3 , available in the online version only). Furthermore, the study by Ramesh et al described a patient in which PSARP approach was intended, but finally a local resection was performed, emphasizing the added value of digital examination preoperatively. 42 Unfortunately, to date, still no consensus is reached as to what technique is most preferred. However, not only operative technique but also comorbidity and potentially associated anomalies or syndromes (e.g., presacral mass in Currarino's syndrome) are of great importance to take into account in choosing the type of surgery. Finally, it is important to choose a technique that fits within the expertise of the surgical team. Therefore, uniform recommendation in this extremely rare disease might not be possible, rather the entire multidisciplinary should outweigh the benefits of each surgical procedure for the individual patient each time taken into account patient-related, doctors-related, and hospital-related factors.

Some techniques are rarely used and are newer, such as magnamosis. Russell et al investigated the use of magnamosis as treatment option for RA describing one patient. Results were promising as no postoperative complications and good functional outcomes were found at follow-up at 4 years in this single patient. 7 This treatment option has been described previously for other congenital malformations, such as esophageal atresia, with varying results. 8 45 Therefore, results should be cautiously interpreted and extrapolated, and the technique should be further assessed as little evidence is available.

In this study, almost 80% of the studies were performed after 2005 which was the year of the publication of the Krickenbeck classification 2 in which RA is classified under “rare-variants” of ARM. In addition, guidelines on RA definitions have been present in literature since 2006, 3 with an update in 2017 depicting a clear differentiation into five types of RA. 4 Still, large heterogeneity was found in RA definitions and, in almost half of the patients, RA was mentioned without further classification or adequate description. 28 30 37 40 Only 14% of the studies were published since the latest 2017 update of which only one study reported an adequate definition. 24 It is important to improve adequate registration, as this is a key to correct interpretation of data and outcomes.

## Strengths and Limitations

This study should be interpreted in light of some strengths and limitations. First, due to a broad search, the risk of missing studies is low. However, to show most current practice, all studies published before January 1, 2000, were excluded, potentially leading to publication bias. In addition, nine authors did not respond to the request for the full text of included studies after title and abstract screening. Furthermore, underreporting of postoperative complications and functional outcomes was present in 27 and 18% of the studies, respectively. Second, solely level IV of evidence studies (i.e., case reports and series) with a small sample size, large heterogeneity (e.g., in patients, procedures, and definitions), and a lot of missing data were included in this study. This hampered the possibility of performing statistical comparisons or a meta-analysis. In addition, no hard conclusions could be drawn. Furthermore, due to the inclusion of solely case reports and series, overall evidence of this systematic review is low. However, all included case reports and series in itself were of moderate to high quality ( Supplementary Table A4 , available in the online version only). Third, almost all studies had a retrospective character leading to inevitable selection and potential information bias. This, however, is the first systematic review on treatment strategies for children with RA that shines light on the various treatment options, but also on the lack of adequate use of definitions and uniform reporting. This is important to identify the optimal treatment strategy for the different types of RA.

Future research into most optimal treatment strategy for RA is necessary. Due to the rare nature of RA, studies investigating this topic entail mostly low levels of evidence. Therefore, most importantly, international centers should collaborate to gain a larger cohort of RA patients and potentially create a Delphi's meeting. Prior to that, uniform definitions of RA should be accepted and core outcome set should be developed (including definitions and optimal measuring instrument for certain (functional) outcomes).

## Conclusion

RA is extremely rare and little is known of the different treatment options. The majority (61/70) of patients reported by the included studies was treated with posterior sagittal or pull-through approach. Little was reported on postoperative complications and functional outcomes at follow-up. Large heterogeneity in surgical techniques and definitions op RA was found. Therefore, no definitive suggestions can be made for the most optimal treatment option for RA but (dis)advantages of the surgical techniques should be outweighed for each individual patient. More importantly, larger cohort studies should be performed to assess the most optimal treatment strategy for children with RA, taking into account accurate reporting of RA type, surgical intervention, postoperative complications, and long-term outcomes.
