# Supplementary material for: Outcomes after Surgical Treatment for Rectal Atresia in Children: Is There a Preferred Approach? A Systematic Review
Source: Eur J Pediatr Surg. 2022 Dec 14;33(5):345–53. doi: 10.1055/s-0042-1758152 (PMC10564564; doi:10.1055/s-0042-1758152)
Supplement: Supplementary file 1 — Supplementary Tables [file 10-1055-s-0042-1758152-s2022066310rev-1.pdf]

Supplementary Table A1 PRISMA 2020 Checklist

Section and topic	Item no.	Checklist item	Location where item is reported
Title			
Title	1	Identify the report as a systematic review.	TP 1
Abstract			
Abstract	2	See the PRISMA 2020 for Abstracts checklist.	Abstract 1
Introduction			
Rationale	3	Describe the rationale for the review in the context of existing knowledge.	M p 2-3
Objectives	4	Provide an explicit statement of the objective(s) or question(s) the review addresses.	M p 2-3
Methods			
Eligibility criteria	5	Specify the inclusion and exclusion criteria for the review and how studies were grouped for the syntheses.	M p 4-6
Information sources	6	Specify all databases, registers, websites, organisations, reference lists and other sources searched or consulted to identify studies. Specify the date when each source was last searched or consulted.	M p 4-6
Search strategy	7	Present the full search strategies for all databases, registers and websites, including any filters and limits used.	SM 1
Selection process	8	Specify the methods used to decide whether a study met the inclusion criteria of the review, including how many reviewers screened each record and each report retrieved, whether they worked independently, and if applicable, details of automation tools used in the process.	M p 4-6
Data collection process	9	Specify the methods used to collect data from reports, including how many reviewers collected data from each report, whether they worked independently, any processes for obtaining or confirming data from study investigators, and if applicable, details of automation tools used in the process.	M p 4-6
Data items	10a	List and define all outcomes for which data were sought. Specify whether all results that were compatible with each outcome domain in each study were sought (e.g. for all measures, time points, analyses), and if not, the methods used to decide which results to collect.	M p 5-6
	10b	List and define all other variables for which data were sought (e.g. participant and intervention characteristics, funding sources). Describe any assumptions made about any missing or unclear information.	M p 5-6
Study risk of bias assessment	11	Specify the methods used to assess risk of bias in the included studies, including details of the tool(s) used, how many reviewers assessed each study and whether they worked independently, and if applicable, details of automation tools used in the process.	M p 4-5
Effect measures	12	Specify for each outcome the effect measure(s) (e.g. risk ratio, mean difference) used in the synthesis or presentation of results.	M p 5-7
Synthesis methods	13a	Describe the processes used to decide which studies were eligible for each synthesis (e.g. tabulating the study intervention characteristics and comparing against the planned groups for each synthesis (item #5)).	M p 6

 © 2022. The Author(s).

Supplementary Table A1 (*Continued*)

Section and topic	Item no.	Checklist item	Location where item is reported
	13b	Describe any methods required to prepare the data for presentation or synthesis, such as handling of missing summary statistics, or data conversions.	M p 5-6
	13c	Describe any methods used to tabulate or visually display results of individual studies and syntheses.	M p 5-6
	13d	Describe any methods used to synthesize results and provide a rationale for the choice(s). If meta-analysis was performed, describe the model(s), method(s) to identify the presence and extent of statistical heterogeneity, and software package(s) used.	M p 5-6
	13e	Describe any methods used to explore possible causes of heterogeneity among study results (e.g. subgroup analysis, meta-regression).	NA
	13f	Describe any sensitivity analyses conducted to assess robustness of the synthesized results.	NA
Reporting bias assessment	14	Describe any methods used to assess risk of bias due to missing results in a synthesis (arising from reporting biases).	M p 5
Certainty assessment	15	Describe any methods used to assess certainty (or confidence) in the body of evidence for an outcome.	NA
Results			
Study selection	16a	Describe the results of the search and selection process, from the number of records identified in the search to the number of studies included in the review, ideally using a flow diagram.	M p 6
	16b	Cite studies that might appear to meet the inclusion criteria, but which were excluded, and explain why they were excluded.	M p 6-7
Study characteristics	17	Cite each included study and present its characteristics.	M p 7-11
Risk of bias in studies	18	Present assessments of risk of bias for each included study.	M p 7
Results of individual studies	19	For all outcomes, present, for each study: (a) summary statistics for each group (where appropriate) and (b) an effect estimate and its precision (e.g. confidence/credible interval), ideally using structured tables or plots.	M p 8-11
Results of syntheses	20a	For each synthesis, briefly summarise the characteristics and risk of bias among contributing studies.	M p 8-11
	20b	Present results of all statistical syntheses conducted. If meta-analysis was done, present for each the summary estimate and its precision (e.g. confidence/credible interval) and measures of statistical heterogeneity. If comparing groups, describe the direction of the effect.	M p 8-11
	20c	Present results of all investigations of possible causes of heterogeneity among study results.	NA
	20d	Present results of all sensitivity analyses conducted to assess the robustness of the synthesized results.	NA
Reporting biases	21	Present assessments of risk of bias due to missing results (arising from reporting biases) for each synthesis assessed.	NA
Certainty of evidence	22	Present assessments of certainty (or confidence) in the body of evidence for each outcome assessed.	NA
Discussion			
Discussion	23a		M p 11-15

(*Continued*)

 © 2022. The Author(s).

Supplementary Table A1 (*Continued*)

Section and topic	Item no.	Checklist item	Location where item is reported
		Provide a general interpretation of the results in the context of other evidence.	
	23b	Discuss any limitations of the evidence included in the review.	M p 11-15
	23c	Discuss any limitations of the review processes used.	M p 11-15
	23d	Discuss implications of the results for practice, policy, and future research.	M p 15
Other information			
Registration and protocol	24a	Provide registration information for the review, including register name and registration number, or state that the review was not registered.	M p 3
	24b	Indicate where the review protocol can be accessed, or state that a protocol was not prepared.	M p 3
	24c	Describe and explain any amendments to information provided at registration or in the protocol.	NA
Support	25	Describe sources of financial or non-financial support for the review, and the role of the funders or sponsors in the review.	M p 16
Competing interests	26	Declare any competing interests of review authors.	M p 16
Availability of data, code and other materials	27	Report which of the following are publicly available and where they can be found: template data collection forms; data extracted from included studies; data used for all analyses; analytic code; any other materials used in the review.	M p 5

Abbreviations: M, manuscript; NA, not applicable; p, page; PRISMA, Preferred Reporting Items for Systematic Reviews and Meta-analyses; SM, supplementary material; TP, title page.
Note: Source: Page MJ, McKenzie JE, Bossuyt PM, Boutron I, Hoffmann TC, Mulrow CD, et al. The PRISMA 2020 statement: an updated guideline for reporting systematic reviews. BMJ 2021;372:n71
For more information, visit: *http://www.prisma-statement.org/*.

 © 2022. The Author(s).

Supplementary Table A2.1 Search strategy in PubMed

Search	Query	Results
#3	#1 AND #2	1,779
#2	"pullthrough"[tiab] OR "pull through"[tiab] OR "Psarp"[tiab] OR "posterior sagittale anorectal plast*"[tiab] OR "Peña Procedure*"[tiab] OR "Pena Procedure*"[tiab] OR ("surgery"[Subheading] OR "Surgical Procedures, Operative"[Mesh] OR "Surgeons"[Mesh] OR "Perioperative Period"[Mesh] OR "Perioperative Care"[Mesh] OR "surg*"[tiab] OR "operat*"[tiab] OR "perioperati*"[tiab] OR "incisi*"[tiab] OR "extracti*"[tiab] OR "excisi*"[tiab] OR "resect*"[tiab] OR "invasive*"[tiab] OR "restorati*"[tiab])	6,093,532
#1	"Rectum atresia*"[tiab] OR "Rectumatresia*"[tiab] OR "Rectal atresia*"[tiab] OR "Rectalatresia*"[tiab] OR "anal atresia"[tiab] OR "analatresia"[tiab] OR "anorectal malformation*"[tiab]	2,915

Supplementary Table A2.2 Search strategy in Embase.com

Search	Query	Results
#4	#3 NOT ('chapter'/it OR 'conference abstract'/it OR 'conference paper'/it OR 'conference review'/it OR 'editorial'/it OR 'erratum'/it OR 'letter'/it OR 'note'/it OR 'short survey'/it)	3,568
#3	#1 AND #2	4,381
#2	'surgery'/exp OR 'surgeon'/exp OR 'perioperative period'/exp OR ('pullthrough' OR 'pull through' OR 'Psarp' OR 'posterior sagittale anorectal plast*' OR 'Peña Procedure*' OR 'Pena Procedure*' OR 'surg*' OR 'operat*' OR 'perioperati*' OR 'incisi*' OR 'extracti*' OR 'excisi*' OR 'resect*' OR 'invasive*' OR 'restorati*'):ti,ab,kw	7,947,356
#1	'anorectal malformation'/exp OR ('Rectum atresia*' OR 'Rectumatresia*' OR 'Rectal atresia*' OR 'Rectalatresia*' OR 'anal atresia' OR 'analatresia' OR 'anorectal malformation*'):ti,ab,kw	7,951

Supplementary Table A2.3 Clarivate Analytics/Web of Science Core Collection

Search	Query	Results
#4	#1 AND #2 and Letters or Notes or Meeting Abstracts or Editorial Materials or Proceedings Papers (Exclude – Document Types)	1,363
#3	#1 AND #2	1,527
#2	TS = ("pullthrough" OR "pull through" OR "Psarp" OR"posterior sagittale anorectal plast*" OR "Peña Procedure*" OR "Pena Procedure*" OR "surg*" OR "operat*" OR "perioperati*" OR "incisi*" OR "extracti*" OR "excisi*" OR "resect*" OR "invasive*" OR "restorati*")	5,676,826
#1	TS = ("Rectum atresia*" OR "Rectumatresia*" OR "Rectal atresia*" OR "Rectalatresia*" OR "anal atresia" OR "analatresia" OR "anorectal malformation*")	2,895

Supplementary Table A2.4 Wiley/Cochrane Library

Search	Query	Results
#3	#1 AND #2	6
#2	("pullthrough" OR "pull through" OR "Psarp" OR "posterior sagittale anorectal plast*" OR "Peña Procedure*" OR"Pena Procedure*" OR "surg*" OR "operat*" OR "perioperati*" OR "incisi*" OR "extracti*" OR "excisi*" OR "resect*" OR "invasive*" OR "restorati*"):ti,ab,kw	34,920
#1	("Rectum atresia*" OR "Rectumatresia*" OR "Rectal atresia*" OR "Rectalatresia*" OR "anal atresia" OR "analatresia" OR "anorectal malformation*"):ti,ab,kw	39

 © 2022. The Author(s).

Supplementary Table A3 Type of definitive surgical correction, type, and definition of rectal atresia

Study	Year	Number of patients	Type of definitive surgery	Type of RA	Definition of RA
Ahn et al[24]	2019	1	Laparoscopic assisted, transanal pull-through	Long gap RA	Blind ending pouch and a significantly distend sigmoid colon + long gap RA approximately 3.5 cm above the anal junction
Gieballa et al[5]	2018	3	Transanal pull-through ($n = 3$)	NS	Failure of contrast to pass more than 2 cm above the anal verge with no fistula + rectal web
					Failure of contrast to pass more than 2 cm above the anal verge with no fistula
					Normal anal opening with obliteration 1cm from the anal verge
Mehmetoğlu[25]	2018	1	PSARP (posterior)	NS	RA without any fistula, and the atretic gap length of the rectum was determined to be ± 1.5 cm
Sharma and Gupta[4]	2017	10	PSARP ($n = 4$), abdominoperineal pull-through ($n = 2$), local excision (transanal ($n = 3$), stricturoplasty ($n = 1$))	Type I: RS	(A) intramural, (B) web with a hole
				Type II	RA with a septal defect
				Type III	RA with a fibrous cord between two atretic ends
				Type IV	RA with a gap
				Type V	Multiple: (A) RA with stenosis, (B) multiple RA, and (C) thickened Houstons valves/multiple rectal stenosis
Braiek et al[26]	2016	1	Local excision (mucosectomy of the web)	Type I RA (mucosal web)	Blind ending anal canal (2-3-cm long) + Dilator abutting the air column in the bowel
Lane et al[27]	2016	1	PSARP (posterior)	NS	Failure to pass a Hegar beyond 2–3 cm
Eltayeb and Shehata[28]	2015	2	Sphincter saving pull-through ($n = 2$)	NS	Not specified
Laamrani and Dafiri[29]	2014	1	PSARP (posterior and anterior)	NS	Atretic inferior rectal segment with a superior rectal pouch and no fistula
Russell et al[7]	2014	1	Magnamosis	RA	Not specified
Hamrick et al[30]	2012	17	PSARP (posterior; $n = 17$)	RA, RS	Not specified
Hamzaoui et al[31]	2012	1	Transanal pull-through	NS	Cordonal RA above the peritoneal reflection + blind ending of the rectum without a fistula
Stenström et al[32]	2011	1	Local excision (endoscopic and transanal)	RA, of septal type	Blind ending dilated intestine, 2 cm from rectum
Hosseini et al[33]	2009	1	Abdominoperineal pull-through	High wind sock RA	Bulging membrane with tiny aperture
Luo et al[34]	2009	3	PSARP (posterior and anterior, $n = 1$), transanal pull-through ($n = 2$)	Type III atresia of the upper rectum	NS
					Gap of 1.5 cm between the proximal gas bubble and distal anal dimple
					Contrast passing proximally to the level of the upper rectum
Lee et al[35]	2007	1	PSARP (posterior and anterior)	NS	Rectourethral fistula and the gap between the two rectal pouches was 0.5 cm
Nguyen and Pham[36]	2007	2	Laparoscopic assisted transanal pull-through ($n = 2$)	NS	Blind ending of the anal canal, 1.5cm above the dentate line. + blind ending of the rectum without fistula

 © 2022. The Author(s).

Supplementary Table A3 (*Continued*)

Study	Year	Number of patients	Type of definitive surgery	Type of RA	Definition of RA
					Blind ending of the anal canal, 2 cm above the dentate line
Ibrahim[37]	2006	4	Transanal pull-through ($n = 4$)	NS	Blind ending of the anal canal, as well as a gap between the rectum and the anal canal
					RA at a distance of 2 cm from the anal verge
					Short distance between the two pouches
					Blind intervening segment of the rectum was short less than 1 cm
Kisra et al[38]	2005	4	PSARP (posterior and anterior, $n = 4$)	NS	Blind ending of the anal canal, as well as a gap between the rectum and the anal canal
					RA at a distance of 2 cm from the anal verge
					Short distance between the two pouches
					Blind intervening segment of the rectum was short less than 1 cm
Kobayashi et al[39]	2005	1	PSARP (posterior and anterior)	NS	Rectum was blind and ectatic and showed the presence of a rectobulbar urethral fistula
Belizon et al[40]	2005	12	PSARP	NS	NS
Saxena et al[41]	2004	1	Local excision (transanal)	NS	Dilated blind upper rectal pouch separated by a 2-mm thin membrane from a well-developed anal canal
Ramesh et al[42]	2002	1	Local excision	NS	Gap of 6 mm between the proximal and distal pouches

Abbreviations: NS, not specified; PSARP, posterior anorectoplasty; RA, rectal atresia; RS, rectal stenosis; SSARP, sphincter saving anorectoplasty.

 © 2022. The Author(s).

Supplementary Table A4.1 JBI quality assessment for case reports

Questions	Studies							
	Ramesh et al[42]	Saxena et al[41]	Kisra et al[38]	Kobayashi et al[39]	Ibrahim[37]	Nguyen and Pham[36]	Lee et al[35]	Luo et al[34]
1. Were patient's demographic characteristics clearly described?	Yes	Yes	Yes	Yes	Yes	Yes	Yes	Yes
2. Was the patient's history clearly described and presented as a timeline?	N.a.	N.a.	N.a.	Yes	N.a.	N.a.	N.a.	N.a.
3. Was the current clinical condition of the patient on presentation clearly described?	Yes	Yes	Yes	Yes	Yes	Yes	Yes	Yes
4. Were diagnostic tests or assessment methods and the results clearly described?	Yes	Yes	Yes	Yes	Yes	Yes	Yes	Yes
5. Was the intervention(s) or treatment procedure(s) clearly described?	Yes	Yes	Yes	Yes	Yes	Yes	Yes	Yes
6. Was the post-intervention clinical condition clearly described?	Yes	Yes	Yes	Yes	Yes	Yes	Yes	Yes
7. Were adverse events (harms) or unanticipated events identified and described?	No	No	Yes	No	Yes	Yes	No	No
8. Does the case report provide takeaway lessons?	Yes	Yes	No	No	Yes	Yes	No	Yes
Total score	19	19	19	18	22	22	16	19
Quality	High	High	High	Moderate	High	High	Moderate	High

Abbreviations: JBI, Joanna Briggs Institute; N.a., not applicable.

 © 2022. The Author(s).

Supplementary Table A4.2 JBI quality assessment for case reports, continuation

	Hosseini et al[33]	Stenström et al[32]	Hamzaoui et al[31]	Russell et al[7]	Laamrani and Dafiri[29]	Lane et al[27]	Braiek et al[26]	Mehmetoğlu[25]	Gieballa et al[5]	Ahn et al[24]
1.	Yes	Yes	Yes	Yes	Yes	Yes	Yes	Yes	Yes	Yes
2.	Yes	N.a.	N.a.	N.a.	Yes	N.a.	N.a.	N.a.	N.a.	N.a.
3.	Yes	Yes	Yes	Yes	Yes	Yes	Yes	Yes	Yes	Yes
4.	Yes	Yes	Yes	Yes	Yes	Yes	Yes	Yes	Yes	Yes
5.	Yes	Yes	Yes	Yes	Yes	Yes	Yes	Yes	Yes	Yes
6.	Yes	Yes	Yes	Yes	Yes	Yes	Yes	Yes	Yes	Yes
7.	No	No	No	No	No	No	No	No	Yes	No
8.	Yes	Yes	No	Yes	No	No	No	No	Yes	No
Total score	21	19	16	19	18	16	16	16	22	16
Quality	High	High	Moderate	High	Moderate	Moderate	Moderate	Moderate	High	Moderate

Abbreviations: JBI, Joanna Briggs Institute; N.a., not applicable.

 © 2022. The Author(s).

Supplementary Table A4.3 JBI quality assessment for case series

Questions	Studies			
	Belizon et al[40]	Hamrick et al[30]	Eltayeb and Shehata[28]	Sharma and Gupta[4]
1. Were there clear criteria for inclusion in the case series?	Yes	Yes	Yes	Yes
2. Was the condition measured in a standard, reliable way for all participants included in the case series?	Unclear	Yes	Yes	Yes
3. Were valid methods used for identification of the condition for all participants included in the case series?	Unclear	Yes	Yes	Yes
4. Did the case series have consecutive inclusion of participants?	Yes	Yes	Yes	Yes
5. Did the case series have complete inclusion of participants?	Yes	Yes	Yes	Yes
6. Was there clear reporting of demographics of the participants in the study?	No	Yes	Yes	Yes
7. Was there clear reporting of clinical information of the participants?	No	Yes	No	No
8. Were the outcomes or follow up results of cases clearly reported?	Yes	Yes	Yes	Yes
9. Was there clear reporting of the presenting site(s)/clinic(s) demographic information?	N.a.	Yes	No	Yes
10. Was statistical analysis appropriate?	Yes	N.a.	N.a.	N.a.
Total score	20	28	22	25
Quality	Moderate	High	Moderate	High

Abbreviations: JBI, Joanna Briggs Institute; N.a., not applicable.

 © 2022. The Author(s).
